# Sperm parameters and mitochondrial DNA sequence variants among patients at a fertility clinic in Ghana

**DOI:** 10.1371/journal.pone.0252923

**Published:** 2021-06-15

**Authors:** Bartholomew Dzudzor, Bismarck Bimah, Vincent Amarh, Augustine Ocloo

**Affiliations:** 1 Department of Medical Biochemistry, University of Ghana Medical School, Korle Bu, Accra, Ghana; 2 Department of Biochemistry, Cell and Molecular Biology, University of Ghana, Legon, Accra, Ghana; Nanjing Medical University, CHINA

## Abstract

**Purpose:**

The present study sought to investigate the common abnormalities and mtDNA mutations in the sperm of Ghanaian men attending the fertility Clinic at the Korle-Bu Teaching Hospital (KBTH). The study therefore provides a baseline data mtDNA mutations in a cross-section of Ghanaian men on referral to the fertility clinic at the KBTH.

**Materials and methods:**

The semen of 55 men attending the fertility clinic were collected from the Urology and the Obstetrics and Gynaecology Departments of the KBTH. Demographic and clinical data were also collected using questionnaires. Semen analyses were performed and were followed by amplification and purification of mtDNA from total DNA extracted from the semen. Sequencing of the mtDNA amplicons was performed using the next generation sequencer (Illumina-MiSeq).

**Results:**

Asthenozoospermia, oligospermia and oligoasthenoteratozoospermia were observed in 1.79%, 5.36% and 28.57%, respectively, of the study participants. There was no association between drinking and/or smoking and history of gonorrhea infection on sperm status/morphology. A total of 785 point mutations were detected in the non-coding control regions, rRNA genes, tRNA genes and the coding regions of the mtDNA samples from the participants. Amongst these mutations, 16 transition mutations were predominantly detected in the mtDNA samples. Missense mutations that were present in only specific sperm abnormalities were identified and they may contribute to infertility in the study population.

**Conclusion:**

The present study has identified various abnormal sperm phenotypes that are prevalent in the study population and provided a baseline data on mtDNA mutations in the spermatozoa of the patients. A wide range of sperm abnormalities were detected in the study population with no association with life style or history of gonorrhea infection. The mtDNA point mutations detected in the selected genes that were analysed were mostly transition mutations. These transition mutations might be critical for the development of abnormal sperm phenotypes underlying male infertility in the Ghanaian population.

## 1. Introduction

According to the WHO, infertility is the failure to achieve conception after a minimum of 12 months of exposure in the absence of known reproductive pathology. Infertility is a global reproductive health issue that affects about 15% of couples worldwide [1]. Approximately 50% of infertility is primarily due to the male and is usually caused by aberrance in sperm motility (asthenozoospermia) or sperm number (oligospermia) [2,3]. Even though sperm can derive its energy needs from glycolysis, sperm motility is heavily dependent on oxidative metabolism occurring in the mitochondrion [4]. Hence, mitochondrial dysfunction could play a significant role in abnormal sperm motility in infertile males.

Each mitochondrion contains covalently closed 16,569 bp DNA molecules encoding for 13, out of 83, subunits of the respiratory chain complexes [1]. Alteration in mitochondrial function due to mutations in either the nuclear or mitochondrial DNA is known to underlie many chronic non-infectious diseases and common metabolic diseases associated with ageing, such as diabetes and cancer. Several studies have highlighted the significant effect of mitochondrial dysfunction on sperm structure and motility [5,6]. Moreover, point mutations and deletions in mtDNA have been observed in many infertile male populations [1,2]. Patients with mitochondrial diseases have shown abnormal sperm structure and function [7]. A relationship between mtDNA haplotype, respiratory function in sperm and asthenozoospermia has been demonstrated and association between ranges of abnormalities in sperm quality, male infertility and a polymorphic variant in the CAG microsatellite repeat of the mtDNA polymerase gene has been reported [8]. Low levels of mtDNA mutations have been identified by several studies across the world in males with infertility and strong correlation between sperm motility and A3243G mtDNA have been observed [9]. However, Palanichamy and Zhang have indicated that more research needs to be done on the identification of other relevant mutations since no single mtDNA can be strongly associated with male infertility based on the current available data on mtDNA [10].

Data on the molecular basis of male infertility in Ghana, and more importantly, mtDNA mutations are scarce. Male infertility in Ghana is usually attributed to reproductive tract infections. However, the involvement of mitochondrial dysfunction in significant percentage of the cases cannot be overemphasized and needs to be investigated. An understanding of the molecular basis and the contribution of mtDNA mutations in male infertility among Ghanaians is very important in the management of the condition. The human mtDNA is maternally inherited [11]. Therefore, common mutations in the mtDNA that are associated with male infertility could serve as biomarkers for screening women that have high risk of transmitting such conditions to their male children. Additionally, the mtDNA is in close proximity to the site of production of free radicals; the absence of histones can increase the risk and rate of mutating mtDNA relative to the nuclear DNA [12].

This study investigated whether common mutations that have been reported to be associated with mitochondrial diseases or male infertility are present among Ghanaian men on referral to the fertility clinic of the Korle-Bu Teaching Hospital. The roles of lifestyle, sperm abnormalities and a reproductive tract infection in male infertility were also reported in this study.

## 2. Methods

### 2.1 Ethical consideration

The study was approved by University of Ghana Noguchi Memorial Institute for Medical Research Review Board (NMIMR-IRB CPN 096/13-14) and the University of Ghana Medical School Review Board (MS-Et/M.11-P3.3/2013-2014). Both oral and written consent were obtained and participants were made to sign an informed consent form to participate in the study. No minors were used in the study.

### 2.2 Sample collection

A total of fifty-five (55) men between the ages of 21 and 50 years, diagnosed with either primary or secondary infertility, were recruited for the study. Men who have undergone vasectomy and other surgical operation in their reproductive system were excluded from the study. Semen samples were obtained from patients by either masturbation or by coitus interruptus. Sample collection and transportation were conducted according to the WHO guidelines [13]; samples were collected in an adjoining room to the laboratory or obtained in the house and transported to the lab within one hour after production. The subjects have also abstained from sexual intercourse for three to five days while avoiding alcohol intake and smoking during this period. Demographic data and other information were collected from volunteers using a structured questionnaire.

### 2.3 Preliminary analyses of semen

Initial macroscopic examination was performed for all the semen samples, according to the WHO guidelines [13]. About 30 minutes after ejaculation, the liquefaction characteristics of the semen were examined and recorded as either complete or incomplete. The colour, volume and pH of the semen were also recorded. Sperm count and morphology, percentage motility, viability, number of pus cell, RBC, and epithelial cells were measured by microscopic examination. The remaining samples were stored in the freezer at -80°C until they were required for DNA extraction.

### 2.4 Isolation of DNA from semen samples

Lymphocytes, RBCs and epithelial cells in the semen samples were removed by the osmotic method, as previously reported [14]. Afterwards, 500 μL of semen was mixed with 10 mL of buffer-1[150 mM NaCl, 10 mM EDTA (pH 8.)]. Sperm cells are able to withstand the chemical conditions in the buffer, unlike the other cells unlike the other cells, which were disrupted. The sperm cells were pelleted by centrifuging at 4500 rpm for 10 min. The supernatant was discarded and the pellet was re-suspended in 0.5 mL of buffer-1 and mixed by votexing. The mixture was centrifuged at 14,500 rpm for 2 min after which the supernatant was discarded. The pellet was treated with 300 μL of buffer-2 [100 mM Tris-HCl pH 8.0, 10 mM EDTA, 500 mM NaCl, 1% SDS and 2% β -mercaptoethanol]. This step was followed by the addition of 20 μL of proteinase–K and the mixture was incubated at 55°C for 2h with occasional inversion of the tube.

The total DNA from the sperm cells were isolated using the QIAamp DNA Mini kit (QIAGEN). An aliquot of 200 μL of buffer AL (lysis buffer) and 200 μL of ethanol were added to the mixture that was incubated from previous step and mixed by vortexing. The mixture was transferred into the QIAamp Mini spin column and centrifuged at 800 rpm for 1 min. The flow-through was discarded and 500 μL of Buffer AW1 was added into the QIAamp Mini column and centrifuged at 800rpm for 1 min to wash the column, after which the flow-through was discarded. The previous step was repeated using Buffer AW2 and centrifuged at 14500 rpm to wash the DNA which are bound on the membrane in the column. The DNA was eluted from the QIAamp Mini spin column by adding 40 μL of Buffer AE, incubating at room temperature for 1 min and centrifuging the spin column at 8000 rpm for 1 min. The final procedure was repeated in order to obtain a volume of 80 μL for each DNA sample. The DNA samples were stored at −20°C until required for amplification.

### 2.5 Amplification of mtDNA

An aliquot of 10 μL of each DNA sample was added to 10 μL of RNAas-free water. A master mix was made by adding 100 μL of the REPLI-g mt Primer Mix to 1.4 mL of the REPLI-g mt Reaction Buffer. The master mix was vortexed and centrifuged at 8000 rpm for 30 sec. Twenty-nine microliters (29 μL of the master mix was added to each diluted DNA sample, vortexed and centrifuged at 8000 rpm for 30 sec. This mixture was incubated at 75°C for 5 min. after which it was allowed to cool to room temperature. After this process, 1 μL of REPLI-g Midi DNA polymerase was added to the mixture and centrifuged at 8000 rpm for 30 sec. This process was also followed by incubation at 30°C for 8 h. The amplified mtDNA were stored at −20°C for further analysis via DNA sequencing.

### 2.6 Purification of amplified mtDNA

The amplified mtDNA were purified using the QIAquick gel extraction kit purchased from QIAGEN^®^. About 200 mg of 1% agarose gel which has the bands containing the mtDNA for each sample was excised with a clean scalpel. An aliquot of 250 μL of the buffer QG was added to the gel and incubated at 50°C for 1 min with occasional shaking to enhance dissolution of the gel. After incubation, the solution was transferred into a QIAquick spin column in a 2 ml collection tube and was centrifuged at 13,000 rpm for 1 min. The flow-through was discarded and the spin column was washed with 500 μL of buffer QG at 13,000 rpm for 1 min, after which the flow-through was discarded. The column was washed with 750 μL of buffer PE by spinning the column at 13,000 rpm for 1 min after which the flow-through was discarded. The spin column was placed into the 2 ml tube and centrifuged again to remove any residue of the buffer PE. About 20 μL of buffer EB (10mM Tris.HCl, pH 8.5) was added to the centre of the QIAquick membrane and centrifuged at 13,000 rpm for 1 min to elute the mtDNA. This last step was repeated to obtain a final volume of 40 μL of mtDNA.

### 2.7 Sequencing and analysis of mtDNA sequence data

Sequencing of the purified mtDNA was done by Inqaba Biotec (South Africa) using the next generation sequencing platform (Illumina-MiSeq). The DNA sequences obtained from the sequencing platform were in the FastQ format and in reads. Quality control (QC) of the sequenced data was checked using FastQC software version 0.10.1. The sequences were mapped to the Revised Cambridge Reference Sequence (rCRS) of the Human Mitochondrial DNA to create BAM files. Quality control was checked again to ensure good quality BAM files were used for downstream analysis. Subsequently, variant calling was performed to identify mtDNA variants in the samples. The variants identified were then annotated and classified into haplogroups using haplogrep. To ensure everything was up to par with sequences, haplocheck was run on the current phylogeny of the mtDNA so as to prevent wrong assignment or misinterpretation and thus rule out contamination from outside mtDNA. A report was generated detailing the variants and their annotation. These sequenced mtDNA data analysis were done using an approach similar to [15].

### 2.8 Statistical analysis

The effect of drinking and/or smoking on sperm morphology was analysed using Chi-square whilst logit regression was performed to determine whether there was any relationship between sperm parameters and history of gonorrhea infection in the study population.

## 3. Results

### 3.1 Demographics and other characteristics of the infertile males

The data for the demographics, lifestyle and clinical history of the infertile study participants are shown in Table 1. The age of the participants ranged from 21 to 50 years, with a mean age of 36.9 years. Majority of the participants were within the age range of 31–40 years (64.15%). The educational level of the participants is also shown in Table 1. Alcohol intake was recorded for 23 (39.63%) participants, with 14 (26.42%) of them taking alcohol occasionally. Seven (7) (12.21%) of the study participants had a history of smoking either marijuana or cigarette. Three of the participants responded to be suffering from hypertension whilst 12 (24.07%) of them responded to have previously been treated for gonorrhoea.

**Table 1:** Demographics, lifestyle and clinical history of the study participants.

Characteristics	Number	Percentage
**Age (Years)**	9	16.98
21–30		
31–40	34	64.15
41–50	10	18.87
**Educational Level**		
Primary	6	11.32
JHS	14	26.41
SHS	16	30.18
Tertiary	12	22.64
None	5	09.43
**Alcohol Intake**		
Daily	4	07.55
Weekly	5	05.66
Occasionally	14	26.42
None	30	60.38
**Smoking history**		
Yes	7	12.21
No	46	86.79
**Lifestyle Disease**		
Hypertension	3	5.56
Gonorrhoea	12	24.07

JHS denotes Junior High School, SHS denotes Senior High School. Data were obtained from 53 out of the 55 study participants; 3 participants did not complete the questionnaire.

### 3.2 Sperm characteristics from semen of the participants

The study population was classified based on their semen parameters using the WHO guidelines [13] and the data obtained is shown in Table 2. The study revealed majority of the participants are abnormospermic (Table 2). Of the 55 study participants, 5 (9.09%) did not have any sperm in their semen (azoospermia), 1 (1.82%) had poor sperm motility (asthenozoospermia), 7 (12.72%) had poor sperm motility with abnormal sperm morphology (asthenoteratozoospermia), 3 (5.45%) were oligozoospermic (low sperm count), 3 (5.45%) had low sperm concentration with poor sperm motility (oligoasthenozoospermia), 16 (29.00%) had low sperm concentration, poor sperm motility and abnormal sperm morphology (oligoasthenoteratozoospermia), 3 (5.45%) had abnormal sperm morphology (teratozoospermia) and 17 (30.91%) had normal semen parameters (normozoospermia).

**Table 2:** Classification of characteristics of spermatozoa in the study participants using the WHO criteria for sperm concentration, motility and morphology.

Nomenclature	Number	Percentage
Asthenozoospermia	1	1.82
Oligozoospermia	3	5.45
Teratozoospermia	3	5.45
Asthenoteratozoospermia	7	12.72
Oligoasthenozoospermia	3	5.45
Oligoasthenoteratozoospermia	16	29.00
Azoospermia	5	9.09
Normozoospermia	17	30.91

Data were obtained from 55 infertile study participants.

### 3.3 Sperm morphology and lifestyle (smoking and/or drinking) among the participants

Out of the total of 53 participants, who answered the questionnaire, 23 (43.40%) consented to either drinking and/or smoking and 30 (56.60%) did not consent to smoking and/or drinking. Table 3 shows results of Chi-square analysis of the sperm morphology among these 23 individuals compared to their counterparts, who did not consent to drinking and/or smoking. Of the 23 participants, who consented to drinking and/or smoking 16 representing 69.67% had abnormospermia compared to 20 representing 66.67% of the 30 participants, who did not consent to drinking and/or smoking. There was no significant difference in the sperm morphology between drinking and/or smoking group and non-drinking and/or non-smoking group.

**Table 3:** Sperm morphology and lifestyle (smoking and/or drinking).

Category of sample	Sperm morphology		Total
	Abnormospermia	Normospermia	
No Drinking and/or Smoking	20	10	30
	(66.67)	(33.33)	(100)
	[55.56]	[58.82]	[56.60]
Drinking and/or Smoking	16	7	23
	(69.57)	(30.43)	(100)
	[44.44]	[41.18]	[43.40]
Total	36	17	53
	(67.92)	(32.08)	(100)
	[100]	[100]	[100]
Pearson chi2 (1) = 0.0502 Pr = 0.823			
Likelihood-ratio chi2 (1) = 0.0503 Pr = 0.823			

Chi-square statistics: Test of significance of the observed differences between sperm parameters of participants that consented to drinking and/or smoking and those who did not. Note: Row percentages in () and column percentages in [].

### 3.4 Sperm status and history of gonorrhoea

Of the 53 participants, who completed the questionnaire, 13 (24.07%) responded to have suffered from gonorrhoea (Table 1). Of the thirteen (13) participants, 9 (69.23%) were abnormospermic and 4 (30.77%) were normospermic (Table 4). All the 9 abnormospermic individuals had low sperm concentration (oligozoospermia), (7) had poor sperm motility (asthenozoospermia) and 8 had abnormal sperm morphology (teratozoospermia). However, there was no statistically significant relationship between sperm status and history of gonorrhea in the study population using Logit regression analysis (result not shown).

**Table 4:** Semen parameters and classification of study population with history of Gonorrhoea.

Parameters	N = 13(%)
**Sperm concentration**	
<20million sperm/mL	4(30.77)
≥20million sperm/mL	9(69.23)
**Sperm Motility**	
≥50% motile	6(46.15)
>50% immotile	7(53.85)
**Sperm morphology**	
>40% with abnormal shape	8(61.54)
≥60% with normal shape	5(38.46)
**Classification**	
Abnormospermia	9(69.23)
Normospermia	4(30.77)

### 3.5 mtDNA mutations in the semen of the study participants

**Fig 1:**
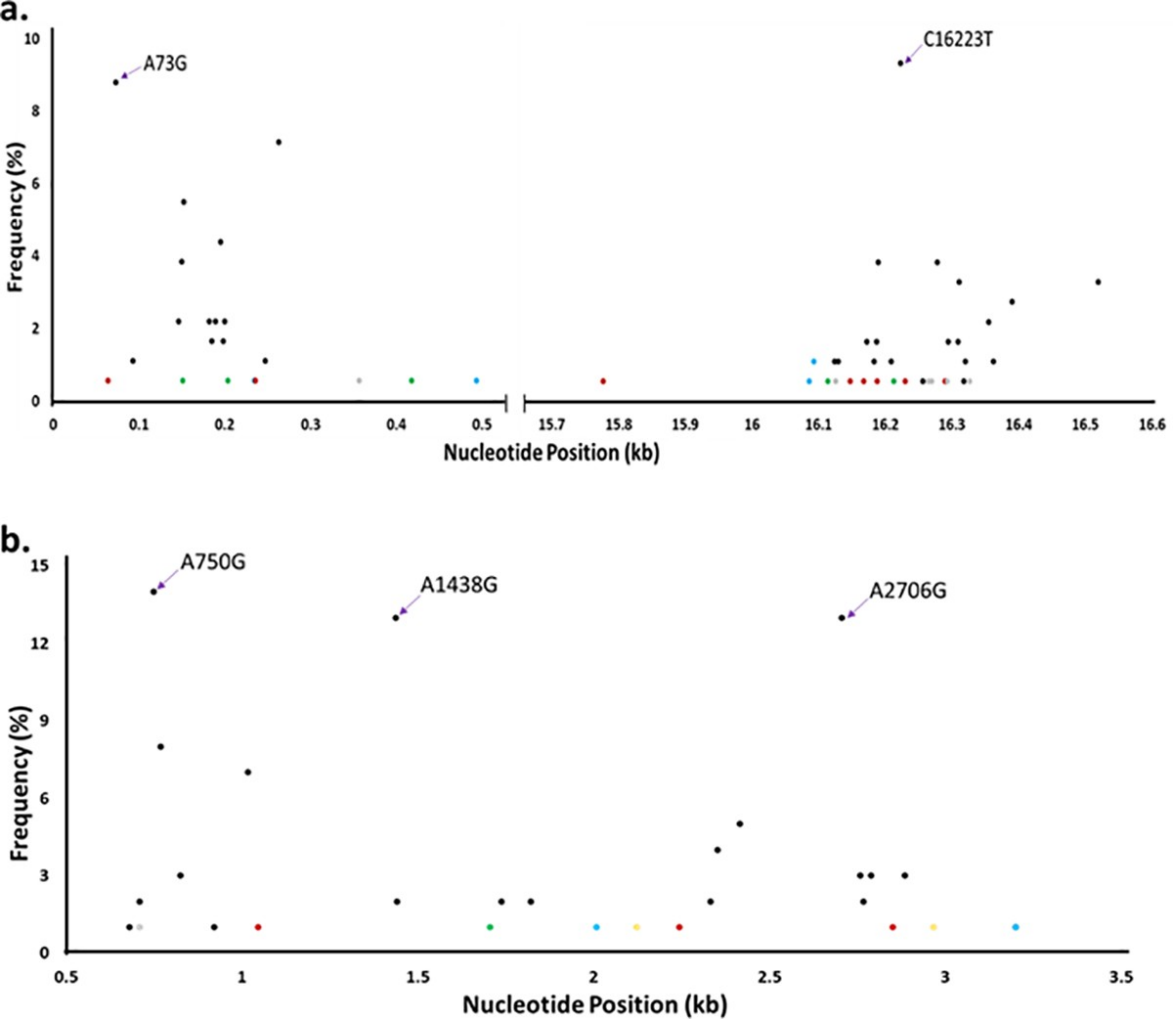
Point mutations at the control regions and rRNA genes of the mtDNA of patients presenting with male infertility. The point mutations detected in only one abnormospermia are represented as oligozoospermia (brown), asthenoteratozoospermia (green), oligoasthenozoospermia (grey) and oligoasthenoteratozoospermia (blue). (a) Point mutations at the control regions. The brown dots are C64T, T236C, G15777A, C16148T, C16168T, C16188G, A16230G and C16290T. The green dots are C151T, T204C, C418T, C16114A and G16213A. The grey dots are A357G, T16126C, C16264T, C16270T, A16293G and C16327T. The blue dots are A235G, C494T, T16086C and T16093C. A73G and C16223T (purple arrows) were the most common mutations in the control region. The total mutations at the control region = 182. (b) Point mutations at the rRNA genes. The brown dots are C1048T, A2245C and A2851G. The green dot is C1706T. The grey dot is T710C. The blue dots are T2010C and T3202C. The yellow dots are A2124G and A2968G. A750G, A1438G and A2706G (purple arrows) were the most common mutations. The total mutations at the rRNAa genes = 100.

**Fig 2:**
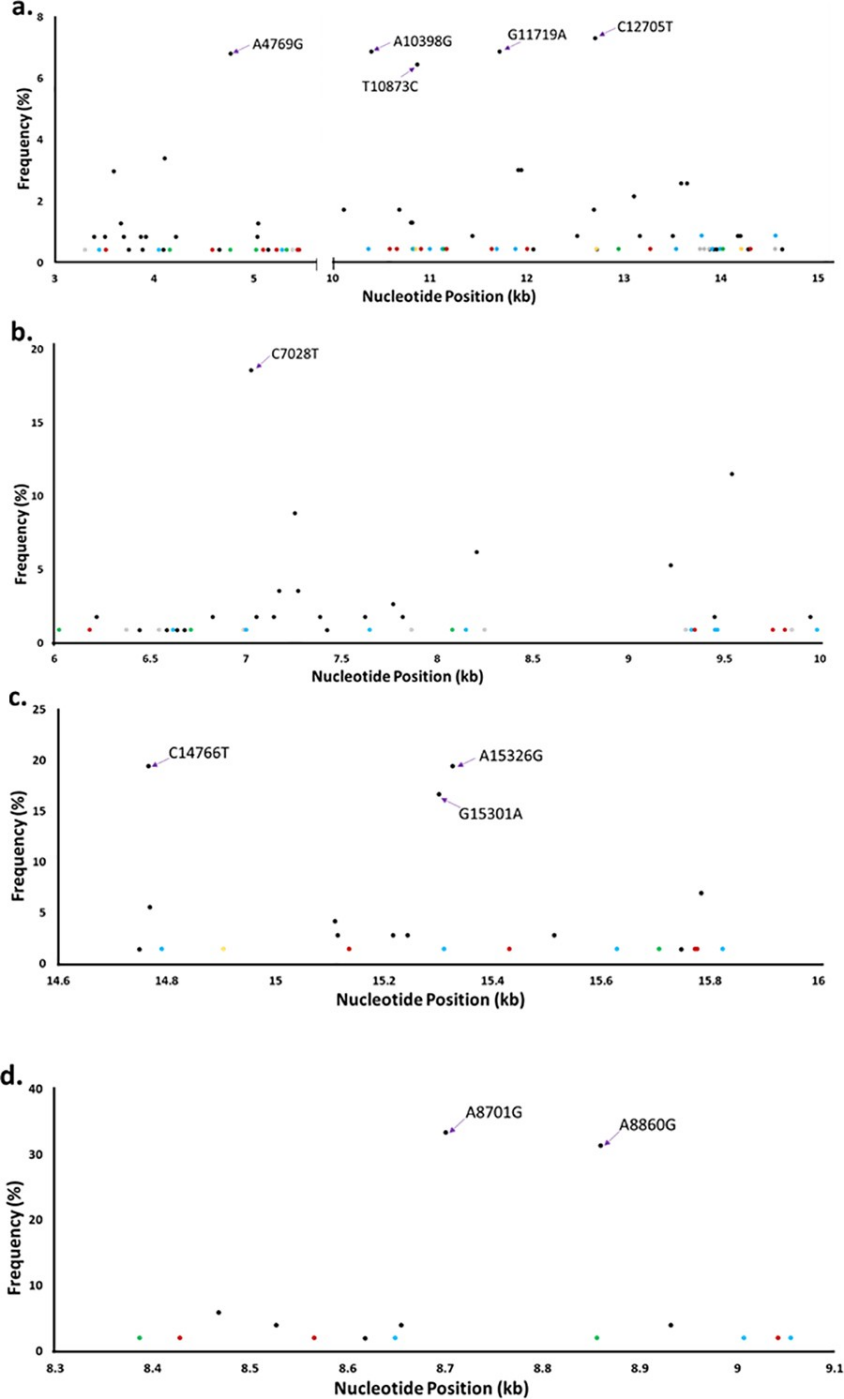
Point mutations at the coding region of the mtDNA of patients presenting with male infertility. The point mutations detected in only one abnormospermia are represented as oligozoospermia (brown), asthenoteratozoospermia (green), oligoasthenozoospermia (grey), oligoasthenoteratozoospermia (blue) and teratozoospermia (yellow). (a) Point mutations at genes encoding subunits of NADH dehydrogenase (ND1-ND6 genes). The brown dots are C3516A, T4586C, T5096C, G5231A, T5442C, G5460A, G10589A, C10664T, T10915C, G11176A, A11641G, G12007A, A13276G and T14308C. The green dots are A4158G, A4767G, C5027T, C5331A, T10828C, G11150A, A12948G and A14022G. The grey dots are T3308C, T5393C, T13789C, C13831A, C13880A and G14560A. The blue dots are C3450T, G4048A, A5285G, G10373A, C10837T, A11002G, C11131T, C11692T, G11887A, A13542G, A13803G, C13914A, C13992T and A14566G. The yellow dots are T10861C, A12723G and T14212C. The underlined nucleotide variants are missense mutations. A4769G, A10398G, T10873C, G11719A and C12705T (purple arrows) were the most common mutations. The total mutations at the ND1-ND6 genes = 235. (b) Point mutations at genes encoding subunits of cytochrome oxidase (CO1-CO3 genes). The brown dots are T6185C, A9347G, G9755A and C9818T. The green dots are G6026A, C6713T and C8080T. The grey dots are T6378C, C6548T, A6989G, C7867T, A8248G, G9300A and A9855G. The blue dots are T6620G, A7004G, C7648T, G8152A, G9329A, G9452A, T9467C and G9986A. The underlined nucleotide variants are missense mutations. C7028T was the most common mutations (purple arrows). The total mutations at the CO1-CO3 genes = 113. (c) Point mutations at genes encoding cytochrome B (CYTB). The brown dots are C15136T, G15431A, G15773A and G15777A. The green dot is A15707G. The blue dots are C14791T, A15311G, T15629C and A15824G. The yellow dot is G14905A. The underlined nucleotide variants are missense mutations. C14766T, G15301A and A15326G (purple arrows) were the most common mutations. The total mutations at the CYTB gene = 72. (d) Point mutations at genes encoding subunits of ATPase (ATPase6/8). The brown dots are C8428T, A8566G and C9042T. The green dots are G8387A and G8856A. The blue dots are A8649G, A9007T and G9055A. The underlined nucleotide variants are missense mutations. A8701G and A8860G (purple arrows) were the most common mutations. The total mutations at the ATPase6/8 genes = 51.

A total of 35 out of the 55 mtDNA samples were sequenced. Sequence data of high quality were received from 28 out of the 35 mtDNA samples submitted for Illumina-Miseq sequencing. The 28 samples consisted of 1 oligozoospermic sample, 2 teratozoozpermic samples, 7 normozoospermic samples, 5 asthenoteratozoospermic samples, 4 oligoasthenozoospermic samples and 9 oligoasthenoteratozoospermic samples (S1, S2 & S3 Tables in S1 File). The sequence data from these samples were used to investigate whether the abnormal sperm phenotypes of the study participants were linked to mutations in their mtDNA. A total of 785 nucleotide variants were identified relative to the nucleotide sequences in the normal reference human mtDNA; 25 of these nucleotide variants were transversion mutations and the remaining 760 variants were transition mutations. The nucleotide variants were detected in the non-coding control regions (mt-DLOOP 2 and mt-DLOOP1), rRNA genes (RNR1 and RNR2), tRNA genes and coding regions. Even though the human mitochondrial genome encodes 22 tRNA genes (of the 37 mtDNA genes), only 18 nucleotide variants were detected in the tRNA genes (S1 Table in S1 File). Moreover, 335 nucleotide variants in the coding region of the mtDNA were silent mutations. The predominant nucleotide variants in the sequence data from the mtDNA samples are shown in Table 5 (and purple arrows of Figs 1 and 2), indicating these mutations are common in the population. These predominant nucleotide variants comprised of 2 point mutations in the non-coding control regions, 3 point mutations in the rRNA genes, 6 silent mutations and 5 missense mutations in the coding region of the mtDNA. The missense mutations resulted in a change of threonine to alanine at positions 59, 112, 114 and 194 of the ATPase (subunit 6), NADH dehydrogenase (subunit 3) and cytochrome b, respectively; an additional change in threonine to isoleucine was detected at position 7 of cytochrome b.

**Table 5:** Predominant nucleotide variants in the mtDNA of the study population.

Genes	Nucleotide position and base change	Change in amino acid and position	Frequency in subjects
12S rRNA	A750G	-	0.58
	A1438G	-	0.54
	A2706G	-	0.54
16S rRNA	A4769G	-	0.67
	C7028T	-	0.88
ND2	A8860G	T112A	0.67
CO1	A8701G	T59A	0.71
ATPase6	A10398G	T114A	0.67
	T10873C	-	0.63
ND3	G11719A	-	0.67
ND4	C12705T	-	0.71
	C14766T	T7I	0.58
ND5	G15301A	-	0.50
CYTB	A15326G	T194A	0.58

12S rRNA = Small ribosomal RNA of the mtDNA; 16S rRNA = Large ribosomal RNA of the mtDNA; ND2, ND3, ND4 and ND5 = subunits 2, 3, 4 and 5 of NADH dehydrogenase; CO1 and CO3 = cytochrome oxidase subunits 1 and 3; ATPase6 = subunit 6 of mitochondrial ATPase; CYTB = Cytochrome b. Frequency in subjects = number of subjects showing the nucleotide variant (n)/total number of subjects whose mtDNA were sequenced (m).

The nucleotide variants at the non-coding control regions, rRNA genes and the coding regions of mtDNA were used to identify point mutations that were unique to each of the abnormal sperm phenotypes. A total of 182 nucleotide variants were detected in the non-coding control regions of the sequenced mtDNA samples (Fig 1A); 12.6% of these nucleotide variants were unique to an abnormal sperm phenotype whiles the A73G and C16223T variants were predominant in the control region of the study participants. Nucleotide variants unique to a sperm abnormality were also detected in the rRNA genes (Fig 1B). Two hundred and thirty-five nucleotide variants were identified in the coding region for NADH dehydrogenase (ND1 –ND6; Fig 2A). The coding regions for cytochrome oxidase (CO1 –CO3), cytochrome b (CYTB) and ATP synthase (ATPase 6/8) had 113, 72 and 51 nucleotide variants, respectively, in the sequenced mtDNA samples (Fig 2B–2D). Notably, point mutations that are unique to each abnormal sperm phenotype were detected in the 13 protein-coding genes of the human mtDNA (coloured dots of Figs 1 and 2). Nucleotide variants that were unique to an abnormal sperm phenotype and caused a change in amino acid in the coding region of the mtDNA are shown in S2 Table in S1 File. The nucleotide sequence data from the mtDNA samples were also used to identify missense mutations that were common to two or three sperm abnormalities with similar phenotypes in the study population.

The affected genes in the coding region of mtDNA containing these common missense mutations include CO1, ND1, ND2, ND6, CYTB, ATPase6 and ATPase8 (S3 Table in S1 File); these common missense mutations might underlie the similar phenotypes in the sperm abnormalities. Collectively, this study provides comprehensive data on the point mutations in the study population. The missense mutations at the coding regions of the mtDNA and the point mutations at the non-coding control regions, rRNA genes and tRNA genes might be contributing factors for the aberrant phenotypes in the sperms of the study participants.

## 4. Discussion

The key function of the mitochondria is to generate ATP. As a result, a cell’s requirement for ATP is proportional to its number of mitochondria. Human mtDNA encodes 13 of the 63 subunits of the electron transport chain and mutations in the mtDNA affects production of ATP and decreases sperm motility since the sperm cells derive most of their energy through oxidative phosphorylation. Studies on mtDNA mutations and their association with male infertility has progressed significantly in the developed countries. However, there is paucity of data in the developing countries, including Ghana. This study has sequenced the mtDNA from males presenting with infertility in Ghana; the data obtained were used to provide information on the prevalent mutations in mitochondrial genome and point mutations that might be associated with the abnormal sperm phenotypes.

Semen analysis identified oligoasthenoteratozoospermia, characterized by low sperm concentration, poor sperm motility and abnormal sperm morphology, as the most common abnomospermia detected in the study participants. This observation is in contrast with data from the study by [16,17], which reported oligozoospermia (low sperm count) and asthenozoospermia (poor sperm motility) as the main cause of male infertility in Ghana and Nigeria, respectively and a meta-analysis of data from 1965 to 2005, reported low sperm concentration as predominant sperm abnormality in Africa [18]. However, our data, indicated that a proportion of the study participants exhibited phenotypes such as absence of sperms in the semen (azoospermia), poor sperm motility (asthenozoospermia), poor sperm motility with abnormal sperm morphology (asthenoteratozoospermia), low sperm count (oligozoospermia), low sperm concentrations with poor sperm motility (oligoasthenozoospermia) and abnormal sperm morphology (teratozoospermia). Thus, our data indicates that the diverse abnormalities of sperm phenotypes in semen are relevant underlying factors in male infertility in Ghana.

Interestingly, individuals with normal sperm phenotypes (normozoospemia) accounted for 30.36% of the study participants diagnosed with primary or secondary male infertility. Similar data have been reported by [19] which showed that 35.36% of their study participants in Indian exhibited normoszoopermic phenotypes. The production of anti-sperm antibodies could interfere with the activity of viable sperms and prevent them from fertilizing the egg [20], thereby contributing to the normozoospemic males presenting with infertility. Moreover, incorrect timing of sexual intercourse by couples can lead to unexplained male infertility when the fertile period of the female is missed [20]. Our data also suggested that infertility was not exclusive to males who had a habit of consuming alcohol and/or smoking. Furthermore, this study showed no association with infertility and history of gonorrhoea infection. This is consistent with a previous finding, which showed no association between *Neisseria gonorrhoea* infection and male infertility [21].

The majority of the mtDNA point mutations identified in this study were transition mutations. The point mutations were identified in the non-coding control regions, rRNA genes, tRNA genes and the 13 protein-coding regions of the human mitochondrial genome. Incidentally, the point mutations at the tRNA genes were very few even though the tRNA genes account for 60% of the genes encoded by the human mitochondrial genome. The frequent occurrence of the T112A, T114A, T7I and T194A missense mutations in the mitochondrial genome of the study participants (both abormospermia and normospermia) indicates that these mutations might have little effect on sperm function and fertility and are rather general mutations. This observation agrees with those reported by [22–24]. The G15301A mutation in the mitochondrial gene encoding cytochrome b, which was also predominantly detected in the study population, has been shown to contribute to unexpected failure during *in vitro* fertilization [25]. Other missense mutations were only present in one sperm abnormality or a group of sperm abnormalities exhibiting similar phenotypes. It can be inferred that these missense mutations uniquely associated with such sperm abnormalities may cause a malfunction in the mitochondrion and consequently, be a determinant in the infertility reported by the patients. For example, the M1V and P136 missense mutations in the ATPase 6/8 genes of the mitochondrial genome may be responsible for the low sperm motility phenotype in the patients presenting with either oligoasthenozoospermia or oligoasthenoteratozoopermia. A comparison of the data from the present study and those from previous studies revealed that mtDNA mutations that had been established to be associated with male infertility were not detected in the present study. The A3243G, T8821C and C11994T mutations in the mt-TL1, ATPase 6 and ND4 genes, respectively, are known mutations in the mtDNA which have been associated with mitochondrial diseases and asthenozoospermia [7,26]; these 3 point mutations were not detected in the mtDNA of the study participants in this study. Nonetheless, other point mutations in the mitochondrial genome which have been implicated in mitochondrial diseases or male infertility, by other studies, were detected in Ghanaian males by this study; an example is the G9055A missense mutation which leads to a change of alanine to threonine at position 177 of the ATPase 6 gene [26].

This study provides a baseline data on point mutations in the mitochondrial genome in men attending a fertility clinic in Ghana. The availability of the mitochondrial genome sequences of the study participants also provides an opportunity for identification of novel mutations that are unique to Ghanaian males diagnosed with primary or secondary infertility.

## 5. Conclusion

The present study has identified various abnormal sperm phenotypes that are prevalent in infertile males in Ghana and provided a baseline data on mtDNA mutations in the spermatozoa of infertile males in Ghana. A wide range of sperm abnormalities were detected in the study population with no association with life style or history of gonorrhea infection. The mtDNA point mutations detected in the selected genes that were analysed were mostly transition mutations. These transition mutations might be critical for the development of abnormal sperm phenotypes underlying male infertility in the Ghanaian population.

## Supporting information

S1 File Contains supplementary tables. (DOCX)

S2 File Mitochondria DNA variant Report 2021–02 -19C3. (XLS)

S1 Dataset Minimal dataset 1. (XLSX)
