# Correction: Sperm parameters and mitochondrial DNA sequence variants among patients at a fertility clinic in Ghana

**DOI:** 10.1371/journal.pone.0256897

**Published:** 2021-08-25

**Authors:** Bartholomew Dzudzor, Bismarck Bimah, Vincent Amarh, Augustine Ocloo

## Notice of Republication

An incorrect version of S1 Dataset was published in error. This article was republished on August 9, 2021 to correct for this error. Please download this article again to view the correct version.
