# Supplementary material for: Sperm parameters and mitochondrial DNA sequence variants among patients at a fertility clinic in Ghana
Source: PLoS One. 2021 Jun 15;16(6):e0252923. doi: 10.1371/journal.pone.0252923 (PMC8205148; doi:10.1371/journal.pone.0252923)
Supplement: S1 File — (DOCX) [file pone.0252923.s001.docx]

**S1 Table: Point mutations at the mtDNA genes encoding tRNA.**

| **Sperm Abnormality** | **Nucleotide mutation** | **Mitochondrial tRNA gene** |
| --- | --- | --- |
| Oligozoospermia | C4312T  C5603T  G7521A | mt-TI  mt-TA  mt-TD |
| Oligoasthenozoospermia | C5601T  T5655C  G7521A | mt-TA  mt-TA  mt-TD |
| Asthenoteratozoospermia | T4370C  T5655C  T5814C  G12236A | mt-TQ  mt-TA  mt-TC  mt-TS2 |
| Oligoasthenoteratozoospermia | T4313C  C5601T  C5663T  G7521A*  T12162C | mt-TI  mt-TA  mt-TN  mt-TD  mt-TH |
| Normozoospermia | A12184G | mt-TH |

*The G7521A point mutation was detected in three different mt-DNA samples from oligoasthenoteratozoospemic patients.

**S2 Table: Missense mutations detected in only one sperm abnormality**

| **Sperm Abnormality** | **Mutations** | | **Affected Gene** |
| --- | --- | --- | --- |
|  | **Nucleotide** | **Amino acid** |  |
| Oligozoospermia | T5442C  G5460A  A8566G  A13276G  G15431A  G15773A  G15777A | F325L  A331T  I14V  M314V  A229T  V343M  S344N | ND2  ND2  ATPase8  ND5  CYTB  CYTB  CYTB |
| Oligoasthenozoospermia | T3308C  G9300A  A9855G  T13789C  C13831A  C13880A | M1T  A32T  I217V  Y485H  L499M  S515Y | ND1  CO3  CO3  ND5  ND5  ND5 |
| Asthenoteratozoospermia | A15707G  A4767G  C5331A  G8387A  G11150A | S321G  M100V  L288I  V8M  A131T | CYTB  ND2  ND2  ATPase8  ND4 |
| Oligoasthenoteratozoospermia | A9007T  G9055A  A15311G  A15824G  G4048A | T161S  A177T  I189V  T360A  D248N | ATPase6  ATPase6  CYTB  CYTB  ND1 |

**S3 Table: Missense mutations detected in two or three sperm abnormalities**

| **Sperm Abnormality** | **Mutations** | | **Affected Gene** |
| --- | --- | --- | --- |
|  | **Nucleotide** | **Amino acid** |  |
| Oligozoospermia & Oligoasthenozoospermia | A7146G | T415A | CO1 |
| Oligozoospermia & Oligoasthenoteratozoospermia | T3866C | I187T | ND1 |
| Oligoasthenozoospermia & Oligoasthenoteratozoospermia | A3505G  A8527G  C8932T | T67A  M1V  P136S | ND1  ATPase8  ATPase6 |
| Asthenoteratozoospermia & Oligoasthenozoospermia | G5046A  T7389C  T14178C | V193I  Y496H  I166V | ND2  CO1  ND6 |
| Oligoasthenozoospermia, Asthenoteratozoospermia & Oligoasthenoteratozoospermia | A14769G | N8S | CYTB |
